# A systematic review and meta-analysis of evidence-based practice and its associated factors among health professionals in Ethiopia

**DOI:** 10.1186/s12913-024-11957-2

**Published:** 2024-11-30

**Authors:** Tolesa Gemeda Gudeta, Ayana Benti Terefe, Girma Teferi Mengistu, Seboka Abebe Sori

**Affiliations:** 1 Department of Nursing, College of Medicine and Health Sciences, Wolkite University, Wolkite, Ethiopia; 2 Department of Midwifery, College of Medicine and Health Sciences, Wolkite University, Wolkite, Ethiopia

**Keywords:** Evidence-based practice, Health professionals, Associated factors, Ethiopia

## Abstract

**Background:**

Evidence-based practice (EBP) entails utilizing the most up-to-date information to inform clinical decisions. Healthcare professionals at all levels are encouraged to integrate the latest research evidence to ensure high-quality care. In Ethiopia, there is a lack of comprehensive, nationally representative data on the prevalence of EBP among healthcare workers. This systematic review and meta-analysis aims to assess the overall prevalence of EBP and its associated factors among health professionals in Ethiopia.

**Methods:**

This systematic review and meta-analysis followed the Preferred Reporting Items for Systematic Reviews and Meta-Analyses (PRISMA) guidelines. The investigators searched electronic databases, including PubMed, Embase, Web of Science, Scopus, Cochrane Library, and Google Scholar, for studies published up to June 1, 2023. Two reviewers independently carried out the extraction of data and evaluation of study quality. We employed STATA version 14 for data synthesis and statistical analysis. The investigators used random-effects and fixed-effects models to calculate pooled odds ratios (ORs) and 95% confidence intervals (CIs) to determine the correlation between dependent and independent factors. The researchers conducted subgroup analysis to explore heterogeneity among the studies included and checked heterogeneity using the I2 statistic. The reviewers assessed publication bias using funnel plots and Egger’s regression test.

**Results:**

The initial search yielded 215 articles, of which 21 met the inclusion criteria. The pooled prevalence of good evidence-based practice among health professionals in Ethiopia was found to be 47.64 [95% CI: 41.33 to 53.96]. Having a masters and above educational status [pooled odds ratio (OR = 3.11; 95%CI: 1.945 to 4.95], good knowledge of EBP [OR: 2.44; 95%CI: 2.14, 2.78], training in EBP [OR = 2.30; 95% CI: 1.91, 2.77], availability of EBP guidelines [OR: 2.05; 95%CI: 1.60, 2.61], internet access [OR: 1.79; 95%CI:1.47, 2.18], availability of free time [OR: 2.05; 95%CI: 1.54, 2.72], presence of administrative support [OR: 1.89; 95%CI: 1.34, 2.67], clinical experience > 5 years [OR: 2.26; 95%CI: 1.54, 3.33], and positive attitude towards EBP [OR: 1.87; 95%CI: 1.53, 2.28] were significantly associated good evidence-based practice in Ethiopia.

**Conclusions:**

Despite the importance of EBP in improving healthcare outcomes, its implementation among healthcare professionals in Ethiopia remains suboptimal. This study also identified modifiable factors associated with EBP adoption. Meanwhile, most of these factors are related to individuals and organizations. Strategies aimed at enhancing access to training and resources and fostering a supportive organizational culture are crucial for promoting EBP uptake and ultimately improving healthcare quality in Ethiopia.

**Supplementary Information:**

The online version contains supplementary material available at 10.1186/s12913-024-11957-2.

## Background

Evidence-based practice (EBP) involves systemically evaluating clinical research to enhance patient care [1], widely recognized as the gold standard in healthcare [2]. It combines evidence, clinical expertise, and patient values to address clinical questions and administrative challenges [3]. EBP reduces costs, improves patient outcomes, and serves as a benchmark for healthcare enhancement. Its efficacy in combating both infectious and chronic conditions is supported by scientific evidence [4]. Furthermore, EBP fosters professional growth, elevates healthcare professionals’ standing in teams, and ensures adherence to current trends, ultimately enhancing patient care [5, 6].

In developed countries like Spain, Ireland, the United States of America, and the United Kingdom, EBP implementation advancing rapidly [7, 8]. However, in Africa, particularly in countries like Ethiopia and Kenya, EBP adoption remains in its infancy stage, with only 42.94% [9] of health professionals in Ethiopia and 8% [10] in Kenya utilizing EBP. In low-income nations where health systems face challenges such as limited standardization and insufficient EBP training, integrating EBP into practice remains challenging [11]. Consequently, patients often receive suboptimal care, leading to longer treatment times, increased costs, and insufficient resource utilization, as healthcare providers often rely on personal experience and outdated guidelines for treatment decisions [12].

The demand for evidence in healthcare decisions is growing in low and middle-income countries due to the heavy burden of diseases. Research indicates that focusing on local contexts and relying on robust scientific evidence for interventions can greatly enhance health outcomes in sub-Saharan Africa [13]. For instance, implementing an evidence-based staffing system in outpatient oncology improved efficiency and satisfaction among both patients and staff within a short period [14]. Similarly, a study in Uganda highlighted the effectiveness of evidence-based practices in enhancing healthcare worker hand hygiene, demonstrating the potential for improving health practices in resource-limited settings [15]. This way, World Health Organization (WHO) advocates for health and social services based on the best research evidence [16].

African healthcare organizations encounter obstacles in embracing in EBP adopting EBP, despite its proven efficacy in enhancing patient outcomes [17]. Commonly cited challenges include interpreting research findings’ relevance to diverse cultural settings, navigating through overwhelming primary research, and the lack of health information technology resources [18]. Limited access to online tools, particularly in remote areas, and insufficient awareness, training, and infrastructure further impede the adoption of EBP among clinicians [19, 20]. These barriers, compounded by healthcare provider shortages and heavy patient loads, underscore the significant challenges in integrating EBP into African healthcare practices [21, 22].

Strategies to promote EBP acceptance of EBP among health professionals require data addressing current obstacles in clinical settings [23]. However, research on EBP in low-income countries is limited. In Ethiopia, despite efforts to strengthen the healthcare system and enhance the quality of care, the extent to which EBP is utilized among health professionals remains unclear. So far, in Ethiopia, a meta-analysis in 2022 pooled data from eight studies [9], but its findings lack generalizability due to few studies and limited geographic representation. Thirteen primary studies were excluded, with reported EBP prevalence ranging from 27.4% [24] to 86% [25]. Updated comprehensive data are crucial to understanding the aggregate prevalence and associated factors. This systematic review and meta-analysis aims to assess the overall prevalence of EBP and its associated factors among health professionals in Ethiopia. Our review may inform targeted interventions to promote evidence-based decision-making in Ethiopia’s healthcare settings.

## Methods

### Protocol and registration

To perform this review, we followed the PRISMA (Preferred Reporting Items for Systematic Reviews and Meta-Analyses) guidelines (Supplementary Table S1). PRISMA has a set of checklists designed to direct the execution and documentation of systematic reviews and meta-analyses, aiming to enhance transparency and precision in reviews across various disciplines, particularly in health sciences. We reviewed the PROSPERO database and the Database of Abstracts of Reviews of Effects (DARE) on the website http://www.library.ucsf.edu/ to ensure that there were no prior or ongoing reviews on the topic. An extensive search on PROSPERO yielded no ongoing or published research on the systematic review and meta-analysis of evidence-based practice and its associated factors among health professionals in Ethiopia. The PROSPERO database officially registered this systematic review and meta-analysis under the ID CRD42024519978 (Supplementary File S1).

### Source of information and search strategy

We designed a comprehensive search strategy to identify relevant studies published in peer-reviewed journals, grey literature, and institutional repositories. The investigation involved searching through databases such as PubMed, Embase, Web of Science, Scopus, and Cochrane Library between May 1 and June 1, 2023, following a predetermined search plan. Additionally, the researchers conducted direct searches on Google Scholar, Google, and online repositories from various universities (including the University of Gondar, Addis Ababa, Jimma, and Haramaya University). The developed search strategy includes a combination of medical subject headings (MeSH) terms and keywords: (“Evidence-based practice” OR “evidence-based utilization”) AND (“Associated factors” OR “determinants”) AND (“Ethiopia”). The researchers also conducted manual searches of relevant articles’ reference lists and consulted experts in the field to discover any further studies within the designated timeframe (Supplementary Table S2).

### Eligibility criteria

To be eligible, studies had to meet the following criteria: (i) assess the prevalence of EBP utilization or associated factors of EBP utilization or both, (ii) assess current research in this area, published between January 2013 and June 1, 2023, (iii) studies conducted among healthcare professionals in Ethiopia, (iv) included only health care professionals, (v) have accessible full-length articles, (vi) studies available in the English language, and (vii) observational studies (used a cross-sectional, case–control, or cohort study design). The exclusion criteria were the complete opposite of the inclusion criteria. Additionally, case reports, editorials, review articles, and conference proceedings were deemed ineligible because of concerns about inadequate information.

### Outcome measurements

This review has two main outcomes. The primary outcome of this systematic review and meta-analysis was the pooled prevalence of good EBP utilization among healthcare professionals in Ethiopia. All the primary studies included assessed the participants’ level of evidence-based practice and classified it into the categories of good and poor evidence-based practice. The results were then organized, analyzed, and presented as the magnitude of evidence-based practice among health professionals. The secondary outcomes were factors associated with the evidence-based practice of health professionals. These were determined using pooled effect sizes (odds ratios) and corresponding 95% confidence intervals based on binary outcomes from the primary studies included.

### Study screening and selection

**Fig. 1:**
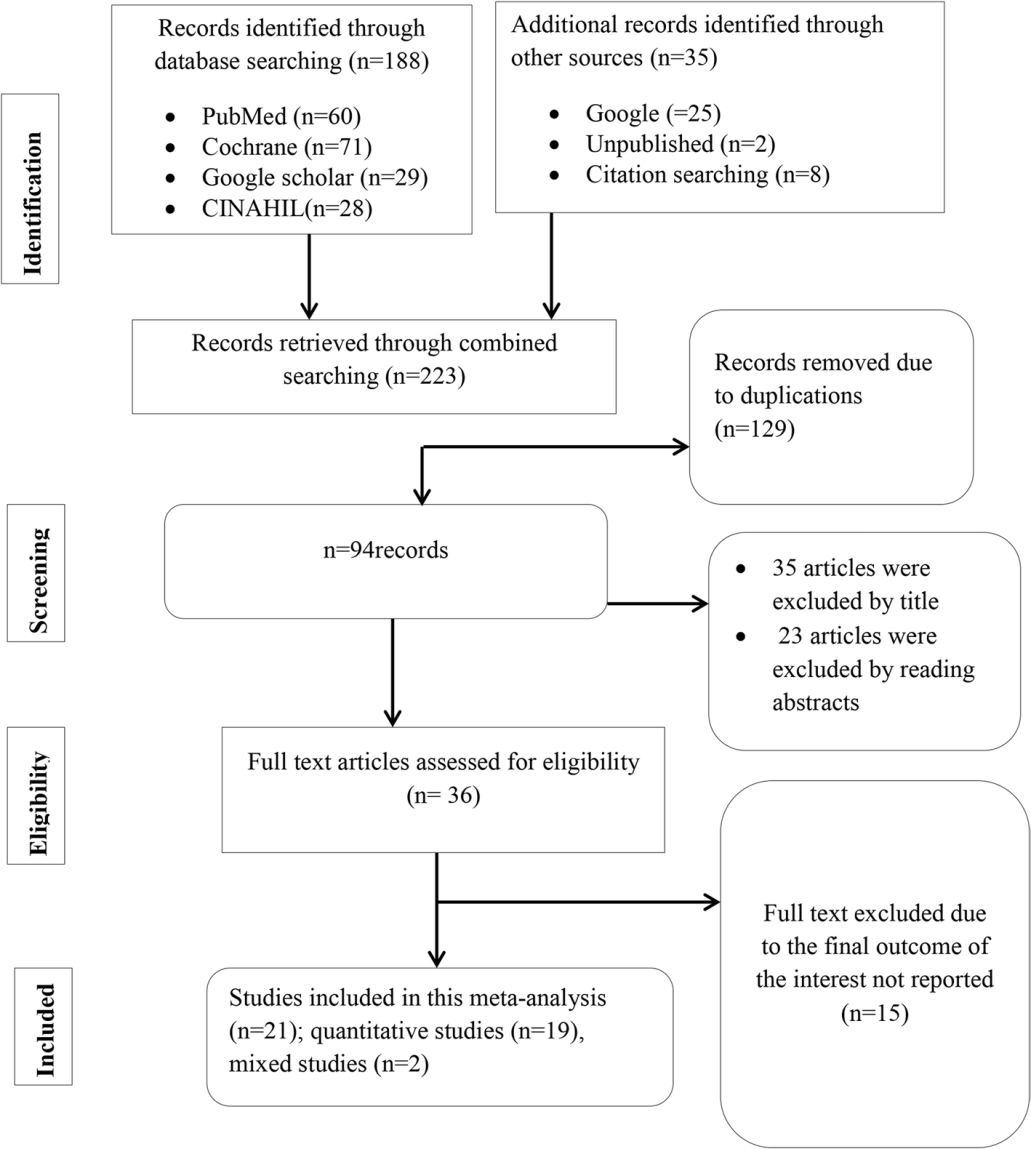
Description of schematic presentation of the PRISMA flow diagram to select and include studies, 2023

Once we imported all the search findings into EndNote X8, we eliminated any duplicate studies. Following this, two authors (TGG and ABT) independently reviewed and assessed the titles and abstracts of the remaining studies based on predefined inclusion and exclusion criteria. The research articles’ titles and abstracts that presented the review’s findings (the evidence-based practice or evidence-based medicine or evidence-based utilization among health professionals AND its associated factors or factors associated with/determinants/predictors/of EBP) were then examined to determine for potential inclusion in the meta-analysis and systematic review. The authors retrieved and evaluated the full text of potentially relevant studies based on their objectives, methods, participants, and results. Any differences in study selection between the two independent reviewers were resolved by consulting more reviewers (SAS and GTM). Here is the PRISMA overview of the study identification and selection process (Fig. 1).

### Data collection process

For the primary outcome, two reviewers (TGG and ABT) independently collected information from the studies included in the analysis using a standardized data extraction form in Microsoft Excel. The data extracted encompassed various aspects of each study, including its characteristics such as primary author, publication year, design, sample size, location, response rate, magnitude of good EBP, and quality assessment score. Accuracy was ensured through double-checking against a data extraction summary (Table 1). For the secondary outcome, (GTM and SAS) gathered quantitative information for the EBP-related factors, such as total sample size (n), EBP magnitude with its 95% CI, and its 95% confidence interval (CI), and compiled using Microsoft Excel 2010 for meta-analysis and synthesis (Supplementary Table S3).

**Table 1:** Descriptive summary of twenty-one studies included in a systematic review and meta-analysis of evidence-based practice and its associated factors among health professionals in Ethiopia, 2023

Authors	Study year	Publication year	Region	Sample size	Response rate (%)	Magnitude of EBP (%)	Study design	Study Population	Study quality
Kassahun et al. [26]	2015	2017	Amhara	217	97.64	38.2	Cross-sectional/ Quantitative study	Health professionals	High
Mitiku et al. [27]	2020	2020	Addis Ababa	398	97	51	Cross-sectional/ Quantitative study	Medical doctor and midwife	High
Debeb Sendekie et al. [28]	2021	2022	Amhara	398	98.0	54.7	Cross-sectional/ Quantitative study	Medical doctor, IESO, and midwife	High
Wodajo S et al. [29]	2022	2022	Oromia	278	100	63.7	Cross-sectional/ Quantitative study	Medical doctor, IESO, and midwife	High
Yideg M [30]	2022	2022	Amhara	422	96.2	44.1	Cross-sectional/ quantitative study	Health professionals	High
Awoke M [31]	2021	2021	Amhara	402	96	55.3	Cross-sectional/ quantitative study	Medical doctor and midwife	High
Megersa Y et al. [32]	2021	2023	Oromia	418	96.4	52.4	Cross-sectional/ Quantitative study	nurses	High
Getenet D et al. [33]	2017	2018	Amhara	415	97.6	55	Cross-sectional/ Quantitative study	health professionals	High
Tadesse B et al. [24]	2017	2017	SNNPR	208	93.3	27.4	Cross-sectional/ Quantitative study	nurses	High
Aynalem ZB et al. [34]	2019	2020	Amhara	684	98.1	55	Cross-sectional/ Quantitative study	nurses	High
Degu AB et al. [35]	2020	2022	Amhara	530	95.7	47	Cross-sectional/ Mixed method	nurses	High
Beshir MA et al. [36]	2015	2017	Amhara	438	98.4	53	Cross-sectional/ Quantitative study	health professionals	High
Dagne et al. [37]	2019	2021	Amhara	826	95.6	34.7	Cross-sectional/ Quantitative study	nurses and midwives	High
Kidist A et al. [25]	2020	2021	Addis Ababa	422	100	86	Cross-sectional/ Mixed method	nurses	High
Wassie et al. [38]	2014	2017	Amhara	177	95.4	40.8	Cross-sectional/ Quantitative study	laboratory professionals	High
Teshager et al. [39]	2017	2019	Oromia	137	90.5	32.3	Cross-sectional/ Quantitative study	Physician	High
Dawit et al. [40]	2015	2018	southwest Ethiopia	333	90.6	39.6	Cross-sectional/ Quantitative study	nurses	High
Bikra et al. [41]	2018	2019	southwest Ethiopia	270	93.7	51.8	Cross-sectional/ Quantitative study	nurses	High
Delelegn et al. [42]	2020	2021	Amhara	423	95.3	48.4	Cross-sectional/ Quantitative study	interns	High
Hadgu et al. [43]	2014	2015	Addis Ababa	217	96.8	57.6	Cross-sectional/ Quantitative study	nurses	High
Alemayehu A et al. [44]	2019	2021	SNNPR	684	98.1	55	Cross-sectional/ Quantitative study	nurses	High

IESO: Integrated emergency surgical officers

### Risk of bias in individual studies

The reviewers utilized the Newcastle Ottawa Quality Assessment Scale (NOS), adapted for cross-sectional research, to methodically evaluate potential bias in individual studies [45]** (**Supplementary Table S4**)**. Each study’s quality was evaluated by two authors (TGG and ABT) based on the study’s (methodological quality, sample selection, sample size, comparability and outcome, and statistical analysis). To mitigate publication bias, we conducted thorough searches employing manual and electronic methods, encompassing diverse research from various settings (both facility-based and community-based studies), incorporating both published and unpublished works. Collaborative efforts among the authors, involving the establishment of a schedule for article selection based on predefined objectives and criteria, quality assessment, ongoing review of the process, and data extraction and synthesis, were crucial in minimizing bias. Additionally, we visually inspected a funnel plot to examine publication bias and assessed the symmetry of the plot using Egger’s Regression Tes [46].

### Data analysis

We conducted data synthesis and statistical analysis utilizing STATA version 14. We utilized a weighted inverse variance random effect model to determine the pooled prevalence of good EBP. Heterogeneity in the studies included was assessed using I-squared and Cochran Q statistics [47]. A value of 75% or above suggested significant heterogeneity [48]. The investigators conducted subgroup analyses based on study region, and sample size and assessed publication bias through funnel plots and Egger’s regression test. We also employed STATA version 14 for data synthesis and statistical analysis concerning factors associated with EBP. Pooled estimates of the association between dependent and independent variables were determined using either a fixed-effects model meta-analysis or a random-effects model meta-analysis. Statistical significance was determined with a p-value below 0.05. The systematic review and meta-analysis results were presented descriptively, along with relevant tables and figures.

## Results

### Article selection

The initial database and manual search yielded a total of 215 studies. After screening titles and abstracts, 36 full-text articles were assessed for eligibility. Finally, 21 studies met the inclusion criteria and were included in the systematic review and meta-analysis (Fig. 1).

### Characteristics of the included studies

We included twenty-one studies in the current systematic review and meta-analysis with 8261 health professionals. All the studies included followed a cross-sectional design across various healthcare settings. Professional disciplines included medicine, nursing, medical laboratory, midwives, public health, and Integrated emergency surgical officers (IESO). Over ninety percent of the research was quantitative, with only two studies utilizing mixed methods [8, 9]. Eleven studies (52%) were conducted in the Amhara region. Almost half (50%) of studies were published between 2021 and 2022 (Table 1).

### The pooled prevalence of good evidence-based practice among health professionals in Ethiopia

**Fig. 2:**
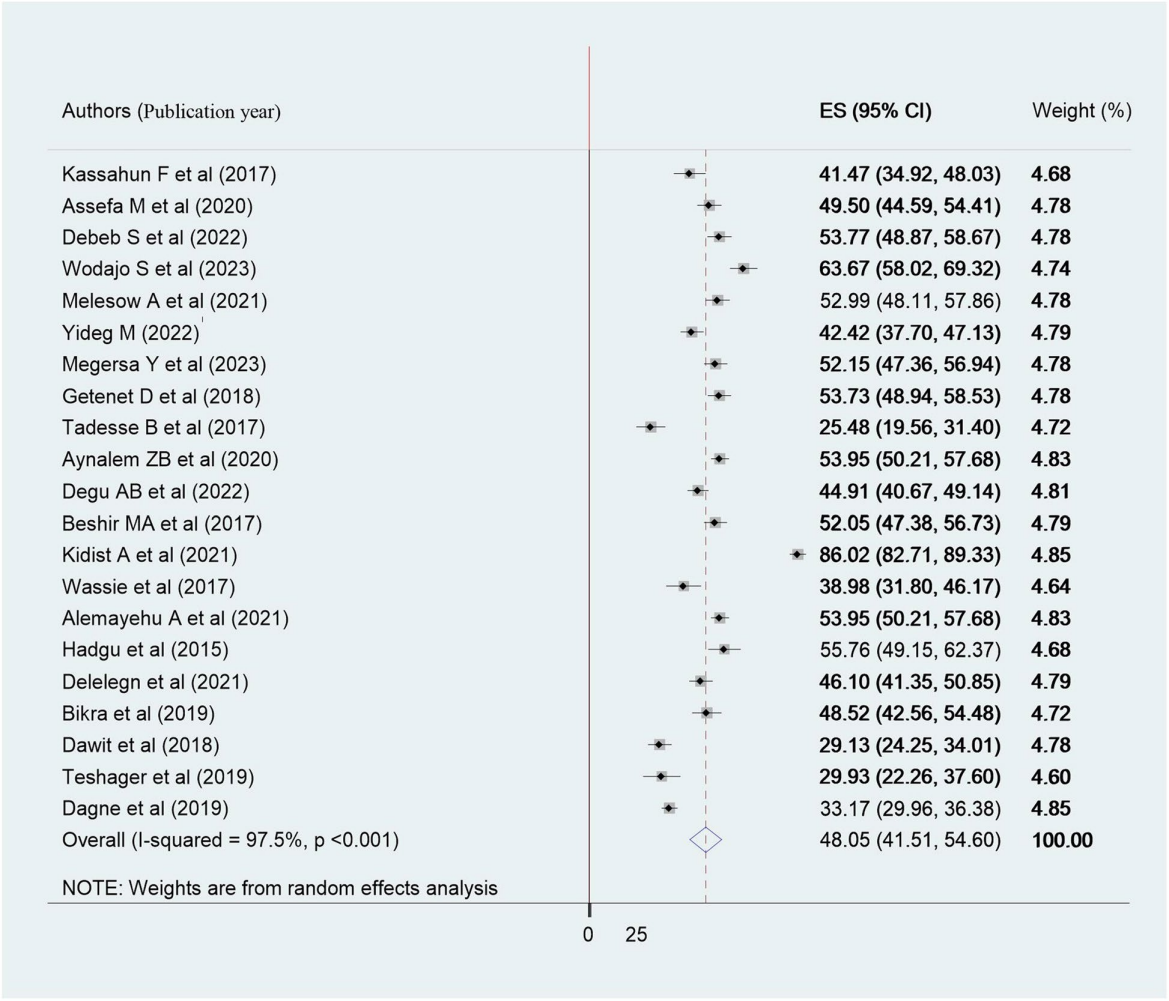
Forest plot showing the pooled prevalence of evidence-based practice among health professionals in Ethiopia, 2023

The prevalence of good evidence-based practice among health care professionals in Ethiopia ranges from 27.4% to 86%% in twenty-one included studies (Table 1). The current meta-analysis demonstrated that the pooled prevalence of good evidence-based practice was 48.05[95%CI: 41.51 to 54.60]. The results of the random effects model showed high heterogeneity among the included studies (*I*^*2*^ = 97.5%, *P* < 0.001)(Fig. 2).

### Subgroup analysis

**Fig. 3:**
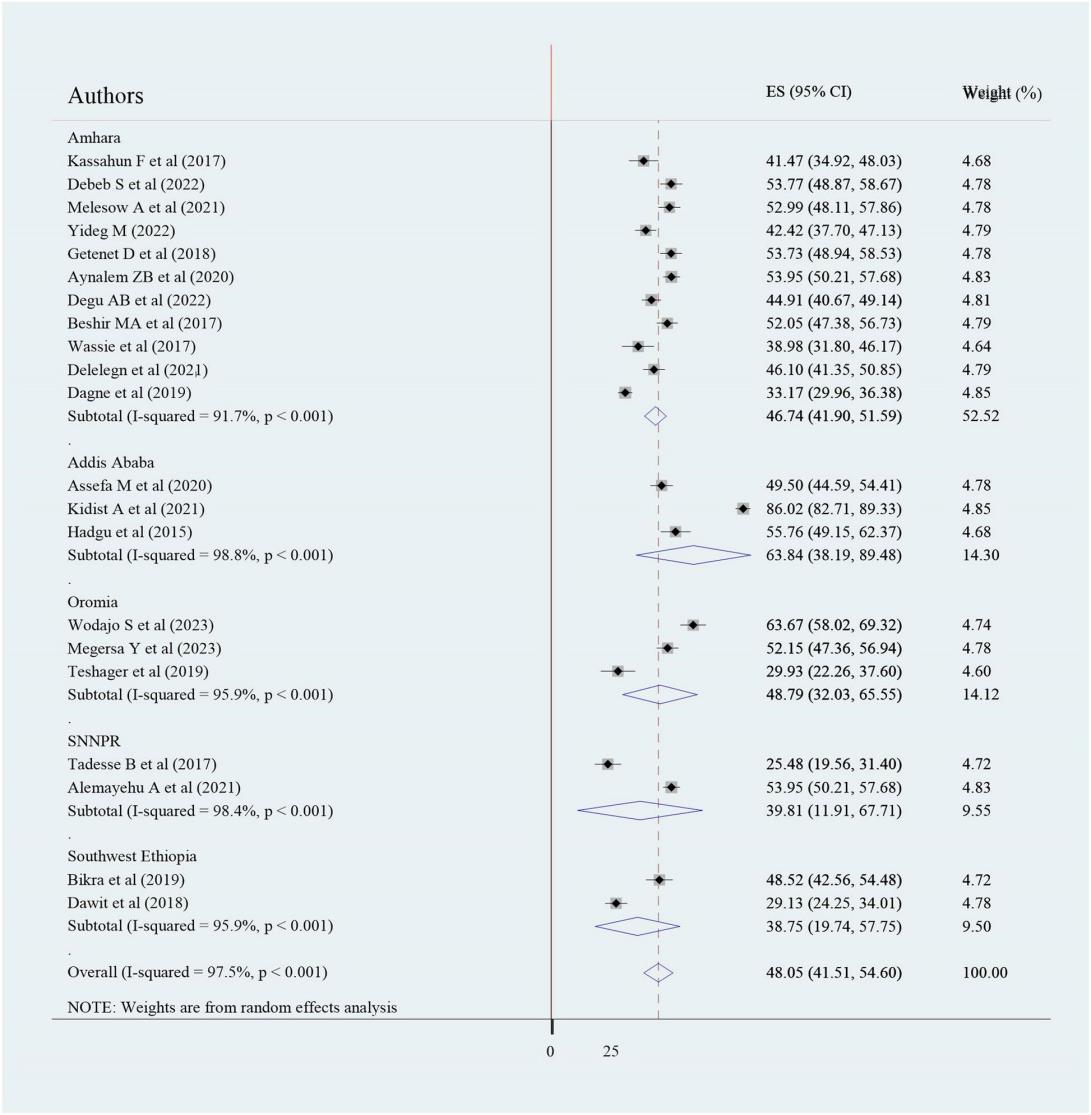
Forest plot showing subgroup analysis of evidence-based practice utilization among health professionals with study region in Ethiopia, 2023

**Fig. 4:**
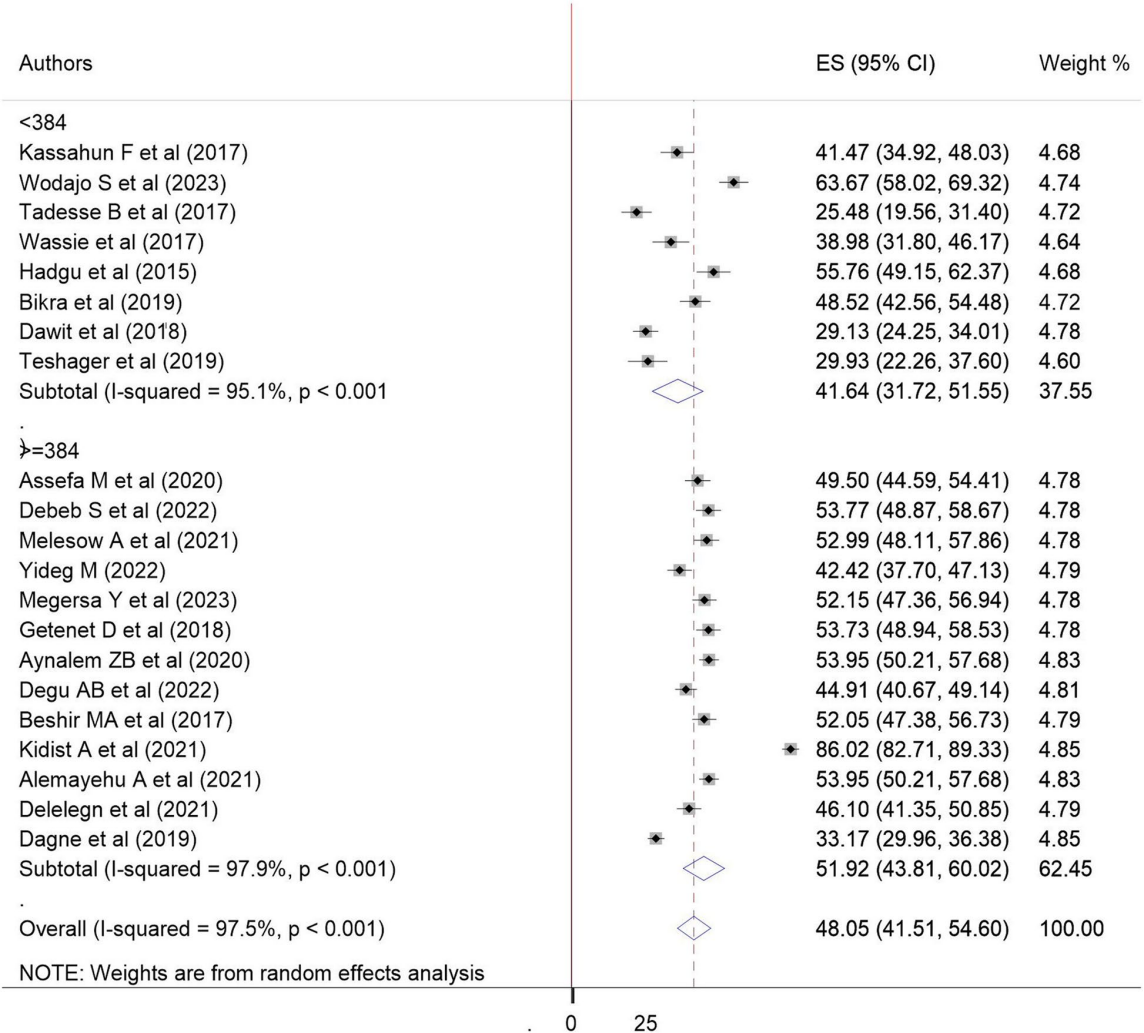
Forest plot showing subgroup analysis of evidence-based practice utilization among health professionals with sample size in Ethiopia, 2023: sample size: < 384, > = 384

This analysis found significant heterogeneity among the included studies (I-squared = 97.5%, *p* < 0.001). We conducted subgroup analyses to determine the source of heterogeneity based on the study region and sample size. The finding of subgroup analysis by the study region depicted that the pooled prevalence of EBP was highest in Addis Ababa (63.84% (95% CI: 38.19–89.48), I2 = 98.8%, *P* < 0.001), whereas the smallest was observed in Southwest Ethiopia (38.75% (95%CI: 19.74–57.75) (Fig. 3). Subgroup analysis based on sample size (< 384 or > = 384) also showed considerable heterogeneity(Fig. 4).

### Small study effect/publication bias

**Fig. 5:**
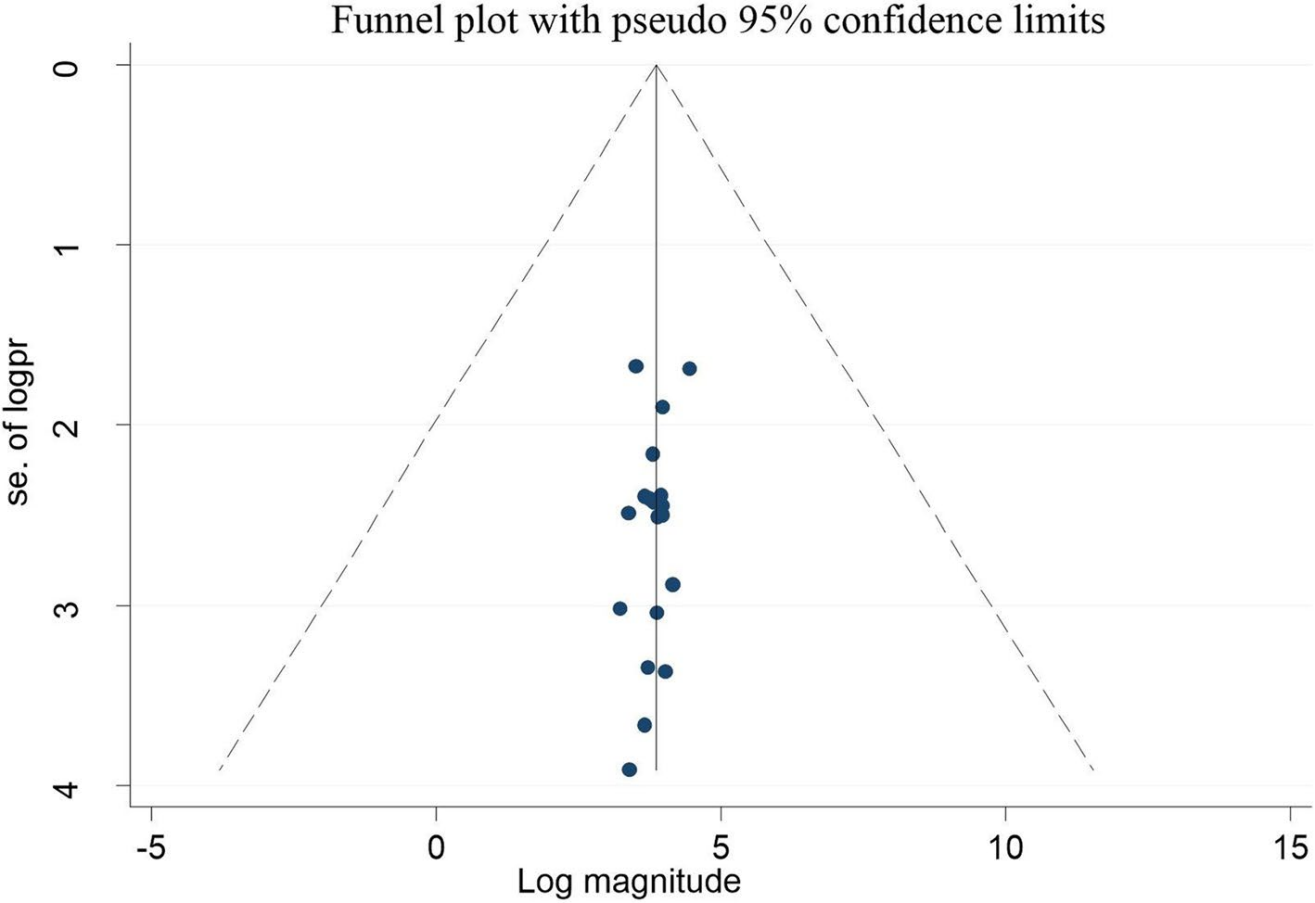
Funnel plot for detecting publication bias on evidence-based practice among health professionals in Ethiopia, 2023

**Fig. 6:**
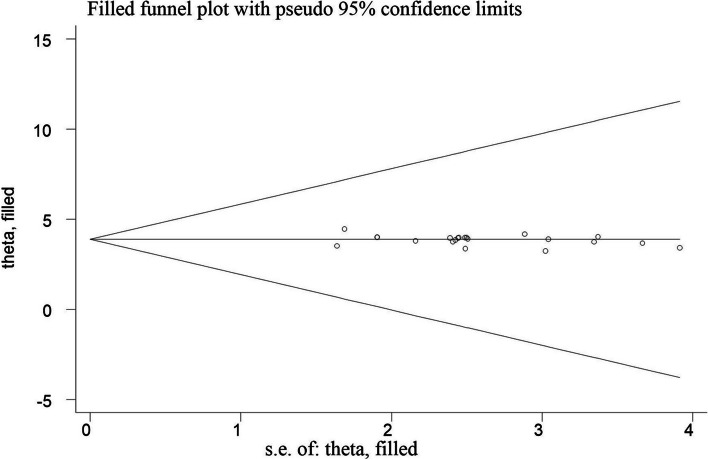
Funnel plots showing the results of trim-and-fill analysis for correcting small study effects

Using Egger’s test and the funnel plot, the reviewers assessed the possibility of publication bias or the effects of small studies among included studies. The funnel plot revealed an asymmetrical distribution, indicating this analysis’s likelihood of publication bias (Fig. 5). Moreover, a small study effect or publication bias was found among the studies included by Egger’s regression test (*p*-value = 0.007). To address this bias, we conducted a “trim-and-fill” analysis to correct for publication bias and generate a funnel plot with symmetrical outcomes. However, despite indications of a small study effect, the outcome remains unchanged in the funnel plot. This bias could be due to the substantial heterogeneity among the included studies, and other factors (Fig. 6).

### Factors associated with good evidence-based practice among health professionals

We identified various factors linked to EBP utilization among Ethiopian health among health professionals in Ethiopia from 21 chosen studies. Most of the factors associated with EBP utilization were individual, organizational, and work environmental factors. The following factors appeared to be significantly associated with EBP utilization among Ethiopian health professionals: level of education, level of health professional knowledge of EBP, access to EBP training, availability of EBP guidelines, internet access, free time availability, administrative support, clinical experience, and attitude toward EBP. Below is an overview of the factors associated with EBP utilization in Ethiopia.

### Association between good evidence-based practice and healthcare professionals’ educational status

As portrayed in Table 2, we included three studies to assess the association between educational status and evidence-based practice. According to the overall OR, healthcare professionals holding a Master of Science degree or higher were nearly three times more likely to have good EBP utilization compared to individuals with educational qualifications below a Master of Science degree [OR = 3.11; 95% CI: 1.945 to 4.95]. A fixed-effect model was employed for analysis since there was no statistically significant heterogeneity among the included studies for this factor (I^2^ = 37.9%, *P* = 0.200).

**Table 2:** Factors associated with good evidence-based practice among health professionals in Ethiopia: a systematic review and meta-analysis, 2023

Variables	Included studies	AOR (95% CI)	Pooled OR (95% CI)	Heterogeneity(I^2^, *p*-value)
Educational level MSc and above	Degu AB et al. [35]	2.15(1.15 to 4.02)	3.11(1.945 to 4.95)	37.9%, 0.200
	Mitiku A [27]	4.09(1.45 to 1.45)		
	Wodajo S et al. [29]	5.75(2.23 to 14.84)		
Knowledge of EBP	Awoke M [31]	4(2.14 to 7.14)	2.44(2.14 to 2.78)	63.1%, 0.001
	Wodajo S et.al [29]	2.95(1.52 to 5.73)		
	Debeb S et.al [28]	2.1(1.3 to 3.38)		
	Kassahun F et.al [26]	5.3(2.01 to 13.9)		
	Mitiku A [27]	2.81(1.79 to 4.37)		
	Yideg M [30]	7.95(4.83 to 13.08)		
	Dagne et.al [37]	3.06(1.6 to 5.77)		
	Alemayehu A et.al [44]	2.04(1.41 to 2.97)		
	Hadgu et.al [43]	3.2(1.5 to7)		
	Delelegn et. Al [42]	1.86(1.22 to2.84)		
	Bikra et.al [41]	2.1(1.12 to 3.89)		
	Wassie et.al [38]	2.22(1.1 to 4.49)		
	Beshir MA et.al [36]	1.61(1.06 to 2.45)		
	Aynalem ZB et.al [34]	2.04(1.41 to 2.97)		
	Megersa Y et.al [32]	1.79(1.13 to 2.82)		
Attitude towards EBP	Kassahun F et. al [26]	3.34(1.3 to 8.6)	1.87(1.53 to 2.28)	73.3%, 0.001
	Wodajo S et.al [29]	3.13(1.59 to 6.16)		
	Awoke M [31]	3.61(2 to 6.45)		
	Dagne et.al [37]	5.02(1.2 to 21.5)		
	Degu AB et.al [35]	1.8(1.24 to 2.62)		
	Alemayehu A et.al [44]	0.94(0.64 to 1.4)		
	Delelegn et. al [42]	2.1(1.3 to 3.2)		
Having trained on EBP	Mitiku A [27]	1.73(1.12 to 2.67)	2.30(1.91 to 2.77)	56.9%, 0.128
	Kassahun F et.al [26]	4.52(1.61 to 12.71)		
	Debeb S et.al [28]	1.81(1.12 to 2.93)		
	Awoke M [31]	2.92(1.3 to 5.67)		
	Yideg M [30]	2.13(1.26 to 3.58)		
	Alemayehu A et.al [44]	3.22(1.96 to 5.31)		
	Aynalem ZB et.al [34]	3.22(1.96 to 5.31)		
	Beshir MA et.al [36]	1.91(1.22 to 2.97)		
EBP guideline availability	Alemayehu A et.al [44]	1.83(1.15 to 2.67)	2.05(1.60 to 2.61)	0.0%, 0.500
	Wassie et.al [38]	2.79(1.41 to 5.52)		
	Aynalem ZB et.al [34]	1.83(1.25 to 2.67)		
	Wodajo S et.al [29]	2.88 (1.46 to 5.7)		
Internet access	Alemayehu A et.al [44]	1.66(1.12 to 2.25)	1.79(1.47 to 2.18)	0.0%, 0.886
	Wassie et.al [38]	2.42(1.12 to 5.29)		
	Beshir MA et. al [36]	1.83(1.19 to2.85)		
	Aynalem ZB et.al [34]	1.66(1.12 to 2.45)		
	Yideg M [30]	2.02(1.25 to 3.27)		
Administrative support	Degu AB et. al [35]	1.89(1.22 to 2.91)	1.89(1.34 to 2.67)	0.0%, 0.001
	Awoke M [31]	1.89(1.08 to 3.35)		
Clinical experience ≥ 5 years	Debeb S et al. [28]	2.13(1.21 to 3.73)	2.26(1.54 to 3.33)	0.0%, 0.771
	Awoke M [31]	2.39(1.4 to 4.05)		
Having sufficient time	Hadgu et.al [43]	7.9(3.5 to 17.6)	2.05(1.54 to 2.72)	83.7%, 0.002
	Delelegn et.al [42]	1.67(1.07 to 2.63)		
	Beshir MA et.al [36]	1.7(1.12 to 2.57)		

### Association between good evidence-based practice and healthcare professionals’ knowledge EBP

As depicted in Table 2, a meta-analysis comprising fifteen studies was conducted to evaluate the correlation between EBP utilization and participants’ level of EBP knowledge. The pooled odds ratio (OR) suggests that health professionals with good knowledge of EBP were 2.44 times more likely to have good EBP compared to those with poor knowledge (OR = 2.44; 95% CI: 2.14 to 2.78). Significant heterogeneity between the included studies was observed (I2 = 63.1%, *P* < 0.001). Therefore, for the study, we used a random effects model.

### Association between good evidence-based practice and health professionals’ attitude towards evidence-based practice

Table 2 illustrates a meta-analysis incorporating seven studies to examine the relationship between health professionals’ attitudes towards evidence-based practice and good evidence-based practice utilization. The pooled odds ratio (OR) indicated that health professionals who had favorable attitudes toward evidence-based practice were approximately two times more likely to have good EBP compared to those who did not [OR = 1.87, 95% CI: 1.53 to 2.28]. The random effects model was used for the analysis since we noted significant heterogeneity among the included studies (*I*^*2*^ = 73.3%, *P* < 0.001).

### Association between good evidence-based practice and healthcare professionals’ EBPs training status

This meta-analysis includes eight articles in all, as Table 2 illustrates. The pooled odds ratio (OR) suggests that health professionals who have evidence-based practice training were 2.30 times more likely to have good evidence-based practice compared to those who did not [OR = 2.30; 95% CI: 1.91 to 2.77]. A fixed-effect model was employed for analysis since there was no statistically significant heterogeneity among the included studies for this factor (I2 = 56.9%, *P* = 0.128).

### Association between good evidence-based practice and the presence of evidence-based guidelines in the workplace

As portrayed in Table 2, a meta-analysis including four studies was performed to assess the association between evidence-based practice and the presence of evidence-based practice guidelines. The overall OR demonstrated health professionals who have standard guidelines at the workplace, were two times more likely to have good evidence-based practice as compared to their counterparts [OR: 2.05; 95%CI: 1.60 to 2.61]. A fixed-effect model was utilized for the analysis since there was no statistically significant heterogeneity among the included studies regarding this factor (*I*^*2*^ = 0%, *P* = 0.500).

### Association between good evidence-based practice and the presence of free time

Three studies were included in the analysis, as shown in Table 2. In the meta-analysis, the pooled odds ratio (OR) suggested that health professionals who have enough time were approximately two times more likely to have good evidence-based practice as compared to their counterparts[OR: 2.05; 95%CI: 1.54 to 2.72]. We used a random effects model due to the substantial heterogeneity detected among the studies included, as indicated by a significant heterogeneity test (I2 = 83.7%, *P* = 0.002).

### Association between good evidence-based practice and internet access

As portrayed in Table 2, we included five studies in the meta-analysis. The overall odds ratio indicated that healthcare workers who have internet access were about 1.79 times more likely to have good evidence-based practice compared to those who did not [OR = 1.79; 95% CI: 1.47 to 2.18]. I-squared = 0.0%, *P* = 0.886 showed no statistically significant heterogeneity among the included studies. As a result, the analysis employed a fixed effect model.

### Association between good evidence-based practice and clinical experience

As depicted in Table 2, a meta-analysis comprising two studies was conducted to evaluate the correlation between evidence-based practice and clinical experience. The pooled odds ratio (OR) indicated that healthcare professionals with > 5 years of clinical experience were 2.26 times more likely to have good evidence-based practice compared to their counterparts [OR: 2.26; 95%CI: 1.54 to 3.33]. A fixed-effect model was employed for analysis since there was no statistically significant heterogeneity among the included studies for this factor (*I*^*2*^ = 0%, *P* = 0.77).

### Association between good evidence-based practice and administrative support

Table 2 illustrates a meta-analysis incorporating two studies to examine the relationship between administrative support and evidence-based practice. In terms of administrative support status, the overall OR indicated health professionals who have administrative support were approximately two times more likely to have good evidence-based practice compared to their counterparts [OR: 1.89; 95%CI: 1.34 to 2.67].

## Discussion

In Ethiopia, the overall pooled prevalence of good EBP was 48.05 (95% confidence interval: 41.51 to 54.59). This outcome agrees with research from studies conducted in Kenya and Zambia, where the prevalence of EBP use was 53.6% and 54%, respectively [49, 50]. However, it is lower than the findings from the study conducted in America, Saudi Arabia, Canada, and Jordan, in which the prevalence of EBP utilization was 67.8%, 59%, 65%, and 60%, respectively [51–54]. On the other hand, this finding is higher than the study done in Australia (33%) [55], Iran (41%) [56], Ghana (25.3%) [57], and Uganda (19%) [58]. This discrepancy may be explained by variations in information technology, internet accessibility, and health professionals’ familiarity with evidence-based practice. Additionally, variations in the clinical infrastructure required for implementing EBP, such as tools, drugs, and support personnel, may account for variations in EBP utilization prevalence.

This review findings revealed a significant correlation between higher education levels (MSc and above) among health professionals and the adoption of evidence-based practice. Healthcare professionals holding a Master of Science degree or higher were nearly three times more likely to have good EBP utilization compared to individuals with educational qualifications below a Master of Science degree. This finding aligns with a study conducted in Jordan and China indicating a positive relationship between education level and evidence-based practice adoption [59–61]. The compared study reported that healthcare professionals with an MSc or higher degree were more likely to implement evidence-based practice than their counterparts. The possible explanation of this finding could be that a higher level of education equips them with enhanced analytical skills and critical thinking abilities, enhancing them to better understand and apply research findings to their clinical practice. Therefore, concerned stakeholders should focus on investing in the education and professional development of healthcare professionals, particularly through advanced degrees, which can enhance their ability to utilize evidence-based approaches, ultimately improving patient care and outcomes.

In our review, healthcare professionals with good knowledge of EBP were 2.44 times more likely to have good EBP compared to those with poor knowledge. By the same token, this finding is similar to a previously conducted systematic review and meta-analysis study in England and other primary studies executed in America and Malawi, which have shown a strong relationship between knowledge and adoption of evidence-based practice [51, 62, 63]. The possible reason for this finding may be healthcare professionals with good knowledge of evidence-based practice recognize its value in improving patient outcomes, enhancing the quality of care, and keeping up with advancement in their field. Moreover, they understand the importance of basing clinical decisions on the best available evidence rather than relying solely on tradition or personal experience. This may imply that efforts should be directed towards providing ongoing education and training programs, particularly for healthcare providers with poor knowledge of EBP to enhance professionals’ understanding and application of evidence-based practice principles, ultimately leading to improved patient care outcomes in the area.

The finding of the current study also indicates that health professionals who had favorable attitudes toward evidence-based practice were approximately two times more likely to have good EBP compared to those who did not. A possible explanation of this finding could be that a positive attitude likely indicates a willingness to engage with new practices and information, including incorporating evidence-based approaches into clinical decision-making. Additionally, those with a favorable attitude may be more motivated to seek out and utilize evidence-based guidelines and resources in their practice. Conversely, those who do not have a favorable attitude towards EBP may be more resistant to change or may not prioritize staying updated with current evidence. Our finding is in line with a systematic review and meta-analysis of evidence-based practice barriers and facilitators in America and another primary study [64, 65]. The compared studies reported that the odds of evidence-based practice are higher among healthcare professionals with a favorable attitude toward it. Hence, our findings may imply the need for implementing educational interventions aimed at promoting positive attitudes toward EBP among healthcare professionals. This may include workshops, seminars, and online courses that highlight the importance of integrating research evidence into clinical decision-making processes, and regular assessment of attitudes towards EBP among healthcare professionals and identify areas for improvement.

Healthcare professionals who have received training in evidence-based practice are 2.3 times more likely to have good evidence-based practice compared to those who did not. The possible reason for this finding may be that evidence-based training equips healthcare professionals with the skills and knowledge to critically appraise and apply evidence in their practice, thus increasing their likelihood of using evidence-based practices compared to those without such training. Our finding is in line with other primary studies conducted in the United Kingdom [66, 67]. In the mentioned study, trained healthcare professionals tend to have a higher prevalence of EBP compared to their untrained counterparts. Hence, our findings may imply the importance of investing in education and training programs for untrained healthcare professionals to ensure the delivery of high-quality, effective, and evidence-based care to patients.

Moreover, healthcare professionals who have standard guidelines at the workplace were two times more likely to have good evidence-based practice as compared to their counterparts. This finding is similar to a previously conducted study on factors associated with evidence-based practice among registered nurses in Sweden [68]. In the mentioned study, the lack of resources such as updated guidelines in the work area is a barrier to EBP use. The possible justification could be that having guidelines readily available can increase confidence in clinical decision-making, reduce uncertainty, and streamline the implementation processes. Furthermore, it fosters a culture that values and prioritizes evidence-based care. Therefore, ensuring healthcare professionals have access to evidence-based guidelines at work is crucial for promoting the adoption of evidence-based practice among them.

The study’s findings show a significant association between internet access and evidence-based practice utilization. Healthcare professionals with Internet access were 1.97 times more likely to have good evidence-based practice than those without Internet access. Our finding is in line with other primary studies conducted in Ghana and Taiwan [57, 69]. In the mentioned study, the presence of the internet is strongly linked to the implementation of evidence-based practices. The possible justification for this statement could be that healthcare professionals with internet success have greater access to up-to-date research, guidelines, and evidence-based resources online, enabling them to more easily incorporate evidence-based practices into their clinical decision-making. Therefore, concerned stakeholders should prioritize providing internet access to their healthcare professionals to enhance evidence-based practice implementation. Additionally, policies promoting Internet access in healthcare settings can facilitate better decision-making and ultimately improve patient outcomes.

In our review, health professionals who have administrative support were approximately two times more likely to have good evidence-based practice compared to their counterparts. This might be because the more administratively supported healthcare providers have better access to continuing education and professional development opportunities related to evidence-based practice, better access to and utilize evidence-based resources and guidelines, better confidence in their ability to implement evidence-based practice, and better communication and coordination within healthcare teams. As a result, they may promote a culture of evidence-based practice. This finding is similar to a previously conducted study on the development and implementation of an inductive model for evidence-based practice in America [70]. Therefore, concerned stakeholders should work on improving administrative support to healthcare professionals as this could improve the implementation of evidence-based practice.

Additionally, another significant factor associated with evidence-based practice is professional experience. Healthcare workers with healthcare professionals > 5 years of clinical experience were 2.26 times more likely to have good evidence-based practice compared to their counterparts. The possible explanation of this finding could be that more experienced healthcare workers often have a deeper understanding of the importance and benefits of evidence-based practice through years of experience and exposure to research. Additionally, experienced workers developed the skills needed to critically evaluate research findings and incorporate them into their practice effectively. Our finding is in line with the study conducted in Iceland [71, 72]. But, our finding is not consistent with the study conducted in Kenya [73]. In the referenced study, novice healthcare workers show a greater tendency to use evidence-based practice compared to their more experienced counterparts. Sociocultural differences can indeed contribute to inconsistencies in beliefs within society.

This study showed that the availability of free time has an association with evidence-based practice. Healthcare professionals who have free time are about 2.05 times more likely to have good evidence-based practice as compared to their counterparts. The possible reason for this finding may be having free time allows professionals to engage more thoroughly in evidence-based practice, such as research and analysis, contributing to better-informed decision-making and practices in healthcare*.* This finding is similar to previously conducted studies in different worlds [74, 75] in which a conducive work setting for implementing evidence-based practice involves having professionals who are not overwhelmed and have sufficient time for evaluating evidence. This discovery indicates that future efforts to enhance evidence-based practice should target organizational obstacles by reducing professional’s workloads, enabling them to thoroughly asses’ and incorporate current research findings into clinical decision-making processes.

### This review has some limitations

All of the studies in this review were cross-sectional, which made it difficult to establish a cause-and-effect relationship between the independent factors and evidence-based practice. The qualities of studies included in this review vary, which may affect the validity and reliability of the overall findings. Research articles published in languages other than English might be missed, resulting in a language bias and an incomplete representation of the available evidence. We also noticed considerable publication bias among the studies. In our meta-analysis, we conducted a “trim-and-fill” analysis to correct for publication bias and generate a funnel plot with symmetrical outcomes. However, despite indications of a small study effect, the outcome remains unchanged in the funnel plot. This bias could be due to the substantial heterogeneity among the included studies, and other factors. The generalizability of the study may be affected by the lack of primary research in some regions, such as Afar, Benishagul Gumuz, and Gambella. Future studies on evidence-based practice among healthcare professionals should take into account these drawbacks.

### Our study also has notable strengths

Although there were some drawbacks, the review had notable strengths. This review revealed significant heterogeneity among the studies, potentially due to variations in the study region, sample size, and other factors. In terms of methodology, most of the studies analyzed had satisfactory quality, and they typically employed sufficient sample sizes, utilized random subject selection, and used validated tools. We carried out an extensive search of both electronic and non-electronic databases to access many studies. This systematic review and meta-analysis followed a rigorous methodology. Lastly, the investigators determined which factors are more strongly associated with optimum dietary practices on which stakeholders should focus their efforts. The current review tried to show factors associated with EBP utilization in theme: individual, organizational, and work environmental related factors.

## Conclusion

This systematic review and meta-analysis provide a comprehensive assessment of EBP implementation and its associated factors among health professionals in Ethiopia. The findings reveal a moderate level of engagement in EBP among health professionals, highlighting both strengths and areas for improvement within the healthcare system. The present review highlighted several factors linked to good EBP. The good thing is that all of these factors are modifiable and the majority of them are related to individual and healthcare settings. Addressing these factors through targeted interventions, including continuous education and training programs, improving access to relevant resources, and fostering a supportive organizational culture, can enhance EBP implementation and ultimately improve the quality of healthcare delivery in Ethiopia. Future research is needed to explore additional factors that may influence EBP uptake and conduct longitudinal studies to establish cause-effect relationships.

## Supplementary Information

Supplementary Material 1.

Supplementary Material 2.

Supplementary Material 3.

Supplementary Material 4.

## Data Availability

No datasets were generated or analysed during the current study.

## Abbreviations

AOR: Adjusted odds ratio
CI: Confidence interval
EBP: Evidence-based practice
PRISMA: Preferred Reporting Items for Systematic Reviews and Meta-Analyses
NOS: Newcastle Ottawa Quality Assessment Scale
OR: Odds ratio
WHO: World Health Organization
