# Supplementary material for: A systematic review and meta-analysis of evidence-based practice and its associated factors among health professionals in Ethiopia
Source: BMC Health Serv Res. 2024 Nov 30;24:1518. doi: 10.1186/s12913-024-11957-2 (PMC11608491; doi:10.1186/s12913-024-11957-2)
Supplement: Supplementary file 2 — Supplementary Material 2. [file 12913_2024_11957_MOESM2_ESM.docx]

**Table S2:** Search method used and Search Results from PubMed on Evidence-Based Practice and Its Associated Factors among Health Professionals in Ethiopia

| **Databases** | | **Searching terms** | **Number of studies** |
| --- | --- | --- | --- |
| PubMed/ MEDLINE | #1 | “Prevalence[MeSH]” OR Magnitude* OR Proportion* | 4,358,269 results |
|  | #2 | **EBP OR “Evidence-Based Practice”** [MeSH] **OR Evidence-Based medicine*[tw] OR evidence-based utilization*[tw]** | 214,710 results |
|  | #3 | **Factor***[tw] **OR associated factor*[tw] OR determinant*[tw] OR factors influencing”** **[tw] OR Predictor*[tw] OR factors associated with*[tw]** | 7,185,016 results |
|  | #4 | **“Health Professional” [**MeSH ] **OR Healthcare Professional*[tw] OR Healthcare provider*[tw] OR** **Clinician*[tw]** | 991,664 results |
|  | #5 | Ethiopia"[Mesh] OR Ethiopia*[tw] | 43,075 results |
|  | #6 | **#1 AND #2 AND #3 AND #4 AND** #5 | 60 results |

**Searches Limited by (Filtered by):**

- **#1 AND #2 AND #3 AND #4 AND #5-** 60 results
- Full texts
- Journal articles
- Studies conducted on Human
- Ethiopia
- English
