# Supplementary material for: A systematic review and meta-analysis of evidence-based practice and its associated factors among health professionals in Ethiopia
Source: BMC Health Serv Res. 2024 Nov 30;24:1518. doi: 10.1186/s12913-024-11957-2 (PMC11608491; doi:10.1186/s12913-024-11957-2)
Supplement: Supplementary file 4 — Supplementary Material 4. [file 12913_2024_11957_MOESM4_ESM.docx]

**Table 3: Quality assessment of articles using Newcastle - Ottawa quality assessment Scale (NOS): (Adapted for cross-sectional studies)**

| **Studies** | **Selection** | | | | **Comparability** | **Outcome** | | **Study quality** |
| --- | --- | --- | --- | --- | --- | --- | --- | --- |
|  | **Representa**  **tiveness**  **(1)** | **Sample**  **size (1)** | **Non-**  **respondents**  **(1)** | **Ascertainment**  **of the exposure**  **(risk factor)**  **(2)** | **The subjects in different outcome groups are comparable, based on the study design or analysis. Confounding factors are controlled (2)** | **Assessment of the outcome**  **(2)** | **Statisti**  **cal test**  **(1)** |  |
| Kassahun et.al | * | * | * | ** | * | ** | * | High |
| Mitiku et.al | * | * | * | ** | * | ** | * | High |
| Debeb Sendekie et al | * | * | * | ** | * | ** | * | High |
| Wodajo S et .al | * | * | * | ** | * | ** | * | High |
| Yideg M | * | * | * | * | * | ** | * | High |
| Awoke M | * | * | * | * | * | ** | * | High |
| Megersa Y et .al | * | * | * | * | * | ** | * | High |
| Getenet D et .al | * | * | * | * | * | ** | * | High |
| Tadesse B et .al | * | * | * | ** | * | ** | * | High |
| Aynalem ZB et.al | * | * | * | ** | * | ** | * | High |
| Degu AB et .al | * | * | * | ** |  | ** | * | High |
| Beshir MA et.al | * | * | * | * | * | ** | * | High |
| Dagne et.al | * | * | * | * | * | ** | * | High |
| Kidist A et .al | * | * | * | ** | * | ** | * | High |
| Wassie et.al | * | * | * | * | * | * | * | High |
| Teshager et. a | * | * | * | * | * | * | * | High |
| Dawit et.al | * | * | * | * | * | * | * | High |
| Bikra et.al | * | * | * | ** | * | ** | * | High |
| Delelegn et. al | * | * | * | * | * | * | * | High |
| Hadgu et.al | * | * | * | ** | * | ** | * | High |
| Alemayehu A et.al | * | * | * | * | * | ** | * | High |

**Descriptions of quality measurement adapted for cross sectional study**

**Selection: (Maximum 5 stars or 5 points)**

1) Representativeness of the sample:

1. Truly representative of the average in the target population. * (all subjects or random sampling): **1 point**
2. Somewhat representative of the average in the target population. * (nonrandom sampling) : **1 point**
3. Selected group of users: **0**
4. d) No description of the sampling strategy: 0

2) Sample size:

1. Justified and satisfactory: **1 point**
2. Not justified: **0**

3) Non-respondents:

1. Comparability between respondents and non-respondents characteristics is established, and the response rate is satisfactory: **1 point**
2. The response rate is unsatisfactory, or the comparability between respondents and non-respondents is unsatisfactory: **0**
3. No description of the response rate or the characteristics of the responders and the non-responders: **0**

4) Ascertainment of the exposure (risk factor):

1. Validated measurement tool : **(2points)**
2. Non-validated measurement tool, but the tool is available or described: **(1 point)**
3. No description of the measurement tool. **0**

**Comparability: (Maximum 2 stars or 2 points)**

1) The subjects in different outcome groups are comparable, based on the study design or analysis. Confounding factors are controlled.

1. The study controls for the most important factor (select one): 1 point
2. The study control for any additional factor: 1 point

**Outcome: (Maximum 3 stars or points)**

1) Assessment of the outcome:

1. Independent blind assessment: **2 points**
2. Record linkage: **2 points**
3. Self-report: **1 point**
4. No description: **0**

2) Statistical test:

1. The statistical test used to analyse the data is clearly described and appropriate, and the measurement of the association is presented, including confidence intervals and the probability level (p value): **1 point**
2. The statistical test is not appropriate, not described or incomplete. **0**

**Note: 1 asterisk or star (*) is equivalent to 1 point**

**Decisions of on the quality of the studies were based on the sum or total score:**

- **High quality studies: >7 points**
- **Moderate quality studies: (five or six points)**
- **Low quality studies: ≤ four points**
